# Sustaining mental toughness under controlling coaching: a moderated mediation model of the coach–athlete relationship and resilience in elite collegiate sports

**DOI:** 10.3389/fpsyg.2026.1858174

**Published:** 2026-06-16

**Authors:** Inwoo Kim, Sujin Kim

**Affiliations:** Department of Sports Science Convergence, Dongguk University, Seoul, Republic of Korea

**Keywords:** coach–athlete relationship, controlling coaching behaviors, mental toughness, resilience, moderated mediation

## Abstract

**Introduction:**

The psychological resilience of elite athletes is often tested by the interpersonal climate established by their coaches. This study aimed to delineate the integrated psychological pathways through which controlling coaching behaviors undermine an athlete’s mental toughness, specifically exploring the interplay between the coach–athlete relationship (CAR) and individual resilience.

**Methods:**

We analyzed data from a sample of 206 elite male university soccer players in South Korea. The hypothesized moderated mediation model was empirically tested using Hayes’ PROCESS macro (Model 15) with a 5,000-sample bootstrap procedure. Structural validity and common method bias were rigorously assessed prior to the main analysis.

**Results:**

Path analysis revealed that while the direct impact of controlling coaching on mental toughness was marginal, its negative influence was primarily channeled through a deteriorated CAR. Crucially, resilience emerged as a potent moderator of the relational conduit; the negative indirect effect of coercive coaching via the CAR was pronounced only among athletes with lower resilience. For those possessing high resilience, this detrimental pathway was effectively neutralized, indicating a significant buffering mechanism.

**Discussion:**

The findings underscore that a high-quality coach-athlete bond is a prerequisite for sustaining mental fortitude, yet this bond is highly vulnerable to controlling interpersonal styles. However, by demonstrating that resilience can decouple relational strain from psychological performance, this study highlights the necessity of resilience-building interventions as a strategic defense against dysfunctional coaching environments.

## 1 Introduction

In contemporary elite sports, the narrowing gap in athletic performance has shifted focus toward the psychological factors required to navigate intensely competitive environments. As the margin for error diminishes, mental toughness defined as the capacity to remain purposeful and efficient under pressure has emerged as a meaningful differentiator of athletic success (Crust, 2007; Liew et al., 2019). This is particularly evident in the collegiate sector, a high-stakes setting where athletes manage a dual-career burden, balancing rigorous academic demands with elite-level training (Stenling and Tafvelin, 2014). For these individuals, the collegiate years represent a period of constant scrutiny, where performance outcomes often dictate future vocational viability. Amidst such chronic pressure and career instability, mental toughness functions as a psychological anchor, supporting an athlete’s commitment and well-being against the susceptibility to burnout (Gustafsson et al., 2017; Sorkkila et al., 2017).

While mental toughness is recognized as a dynamic resource, it is significantly influenced by environmental factors, particularly the interpersonal style of the coach. Guided by Self-Determination Theory (SDT), scholarly discourse outlines two contrasting interpersonal styles: autonomy-supportive coaching and controlling coaching (Deci and Ryan, 1985; Ryan and Deci, 2017). Although extensive literature highlights the psychological benefits of autonomy-supportive environments, the intensely competitive and result-oriented nature of elite sports often renders controlling coaching strategies pervasive, and at times perceived as necessary for maintaining structural discipline (Morbée et al., 2020). Grounded in a differentiated approach, this controlling style operates through four distinct practices: intimidation, negative conditional regard, controlling use of rewards, and excessive personal control (Bartholomew et al., 2010). Despite their prevalence in high-performance contexts, these restrictive behaviors act as potent environmental stressors that can compromise an athlete’s psychological fortitude (Bartholomew et al., 2011; Stebbings et al., 2012). Because the long-term impact of such a restrictive style on mental toughness remains theoretically fragmented, it is valuable to examine the underlying relational pathways through which these coaching practices operate.

These environmental pressures are most immediately manifested through the coach–athlete relationship (CAR), a multidimensional bond encompassing closeness, commitment, and complementarity (Jowett and Ntoumanis, 2004; Wekesser et al., 2021; Longakit et al., 2024). Together, these facets function as a relational lens through which a coach’s interpersonal style is interpreted (Jowett, 2017). From a theoretical standpoint, this dyadic bond serves as a critical parallel mediating mechanism within the athletic environment. Interpersonal stressors like controlling coaching can increase friction and weaken these specific relational facets, potentially lowering the athlete’s capacity for effective self-regulation (Jowett, 2003). Consequently, evaluating how these discrete relational pathways are undermined is essential for understanding the underlying mechanisms that shape or potentially compromise mental toughness.

The impact of such interpersonal stressors is not uniformly distributed across all athletes, indicating that specific boundary conditions modulate these associations. Within this framework, resilience conceptualized as a dynamic process of positive adaptation in the face of adversity functions as a psychological buffer that interacts with the pathways of environmental influence (Luthar et al., 2000; Masten, 2011). Athletes with higher resilience tend to possess superior self-regulatory capacities and cognitive flexibility, enabling them to neutralize the negative consequences of a compromised coach–athlete relationship (Fletcher and Sarkar, 2013; Sarkar and Fletcher, 2014). By facilitating positive reappraisal, resilience may attenuate the link between relational strain and the depletion of mental toughness, effectively supporting an athlete’s psychological fortitude even when the relational foundation is weakened.

To date, research has often examined environmental influences, relational mechanisms, and individual traits in isolation, leaving a gap in the holistic understanding of how mental toughness is maintained. The present study addresses this gap by proposing and testing an integrated moderated mediation model. The primary objective is to examine whether the mediated relationship between controlling coaching and mental toughness through the coach–athlete relationship is moderated by an athlete’s level of resilience. Moreover, to fully capture the psychological dynamics involved, this study extends the integrated model to the sub-dimension level. Grounded in recent differentiated frameworks (Barcza-Renner et al., 2016; Morbée et al., 2020; Longakit et al., 2024), we evaluate how the four discrete facets of controlling coaching selectively undermine the three relational pathways, and how resilience functions as a conditional firewall across these specific paths. This comprehensive approach aims to clarify the mechanisms through which mental toughness is sustained or compromised, offering a more precise framework for psychological risk management in elite collegiate sports.

### 1.1 Controlling coaching and mental toughness

The development of mental toughness is deeply rooted in the motivational climate established by the coach. This dynamic is elucidated through Self-Determination Theory (SDT), which posits that coaching styles influence athlete outcomes by either facilitating or thwarting innate psychological needs (Deci and Ryan, 2000; Ryan and Deci, 2017). While autonomy support is linked to positive growth, the “dark-side” of coaching specifically controlling behaviors remains an area requiring differentiated empirical examination (Mageau and Vallerand, 2003). Bartholomew et al. (2010) conceptualized controlling coaching as a multidimensional construct comprising four discrete facets: intimidation, negative conditional regard, controlling use of rewards, and excessive personal control.

Recent scholarly reviews underscore that coaching behaviors are multifaceted, and their associations with athlete outcomes are not uniform across sub-dimensions (McShan and Moore, 2023). For instance, whereas some facets may be perceived as necessary for structural discipline, others such as negative conditional regard are consistently linked to decreased psychological well-being (Bartholomew et al., 2011). These distinct facets of controlling coaching act as potent environmental stressors that deplete the psychological resources required for sustained mental toughness (Stebbings et al., 2012). By treating these sub-dimensions as primary constructs, we can identify which specific controlling practices pose the greatest threat to an athlete’s mental fortitude. Consequently, this study operationalizes controlling coaching through its discrete facets to clarify the behavioral pathways that directly compromise mental toughness.

Hypothesis 1: Athletes' perceptions of controlling coaching behaviors will be negatively associated with their mental toughness.

Hypothesis 1-1: The specific facets of controlling coaching—(a) intimidation, (b) negative conditional regard, (c) controlling use of rewards, and (d) excessive personal control—will be negatively associated with mental toughness.

### 1.2 The mediating role of the coach–athlete relationship

The impact of controlling coaching on mental toughness is likely transmitted through the coach–athlete relationship. According to the 3 + 1Cs model, this bond is defined by closeness, commitment, and complementarity (Jowett, 2017). Based on Interdependence Theory, the psychological state of an athlete is significantly influenced by these dyadic interactions (Kelley and Thibaut, 1978). Controlling coaching behaviors function as relational stressors that increase friction and diminish trust (Jowett, 2003). Specifically, controlling behaviors such as intimidation or negative conditional regard may uniquely erode relational quality. For instance, coercive practices can diminish an athlete’s sense of ‘complementarity’ by restricting their autonomy within the dyad, while ‘negative conditional regard’ may weaken the affective bond of ‘closeness’ and the cognitive intention of ‘commitment’ to the relationship (Jowett, 2017; McShan and Moore, 2023). When these specific relational facets are compromised, the athlete’s capacity for effective self-regulation and psychological fortitude is depleted (Gucciardi et al., 2015; Jowett, 2003). Therefore, we hypothesize that the discrete facets of controlling coaching exert their influence by undermining these specific relational dimensions, which in turn leads to the depletion of mental toughness.

Hypothesis 2: Coach–athlete relationship (CAR) will mediate the relationship between perceived controlling coaching behaviors and mental toughness.

Hypothesis 2-1: The specific sub-dimensions of the CAR—(a) closeness, (b) commitment, and (c) complementarity will mediate the relationship between the discrete facets of controlling coaching and mental toughness.

### 1.3 The moderating role of resilience

Interpersonal stressors do not exert a uniform impact on all athletes, suggesting that specific boundary conditions modulate the strength and direction of these associations (Jowett, 2017; Baron and Kenny, 1986). Identifying these moderating factors is essential for understanding why certain athletes maintain their mental fortitude even when their primary support systems or environmental conditions are compromised (Gucciardi et al., 2011; Windle, 2011). Within this framework, resilience emerges as a dynamic process of positive adaptation that functions as a psychological buffer against external pressures (Luthar et al., 2000; Masten, 2011). In elite sport settings, resilience involves the effective mobilization of psychological resources to withstand and even thrive amidst sustained adversity (Fletcher and Sarkar, 2013; Galli and Vealey, 2008). Consistent with the need for differentiated examination of coaching facets and relational dynamics (McShan and Moore, 2023), resilience is hypothesized to modulate the pathways within our integrated model. Highly resilient athletes possess superior self-regulatory capacities and cognitive flexibility (Sarkar and Fletcher, 2014). These internal assets allow them to engage in positive reappraisal, viewing a coach’s specific controlling behaviors such as intimidation or excessive personal control as manageable challenges rather than threats to their self-identity (Fletcher and Sarkar, 2013; Hu et al., 2015). Furthermore, resilience serves as a “conditional firewall” regarding the coach-athlete relationship (CAR). Even when relational facets like ‘complementarity’ or ‘commitment’ are weakened (Jowett, 2003), resilient individuals can utilize compensatory strategies to maintain their psychological state (Anyan and Hjemdal, 2016). By analyzing these interactions at the sub-dimensional level, we can pinpoint whether resilience specifically buffers against relational strain or if its effect is pervasive across different manifestations of coaching control.

Hypothesis 3: resilience will moderate the direct relationship between perceived controlling coaching behaviors and mental toughness, such that the negative association will be attenuated for athletes with higher levels of resilience.

Hypothesis 3-1: Resilience will moderate the direct association between the specific facets of controlling coaching and mental toughness.

Furthermore, resilience is expected to intervene in the relationship between the coach–athlete relationship and mental toughness. Even when the quality of the dyadic bond is weakened characterized by diminished trust, lack of commitment, or interpersonal friction (Jowett, 2003; Jowett and Cockerill, 2003) highly resilient individuals can utilize compensatory strategies to maintain their psychological state. Resilience facilitates a buffering effect where internal adaptive resources reduce the impact of relational strain on performance-related outcomes (Anyan and Hjemdal, 2016; Rutter, 2012). By maintaining a sense of agency and purpose despite a compromised relational foundation, resilient athletes can effectively sustain their mental toughness even in the absence of optimal social support (Gucciardi et al., 2015; Wagstaff et al., 2017).

Hypothesis 4: resilience will moderate the relationship between the perceived CAR and mental toughness, such that the positive association will be strengthened (or the impact of a weakened relationship will be buffered) for athletes with higher levels of resilience.

Hypothesis 4-1: Resilience will moderate the relationship between the CAR sub-dimensions (closeness, commitment, and complementarity) and mental toughness.

By simultaneously modulating these direct and indirect pathways, resilience serves as a significant boundary condition for the entire motivational process. This integrated perspective suggests that the extent to which controlling coaching erodes mental toughness through the quality of the coach–athlete relationship depends heavily on the athlete’s internal capacity for positive adaptation (Kim et al., 2026; Morgan et al., 2013). This approach accounts for the complex interplay between the external social context and the individual’s psychological resilience in maintaining mental fortitude (Galli and Gonzalez, 2015).

**Figure 1:**
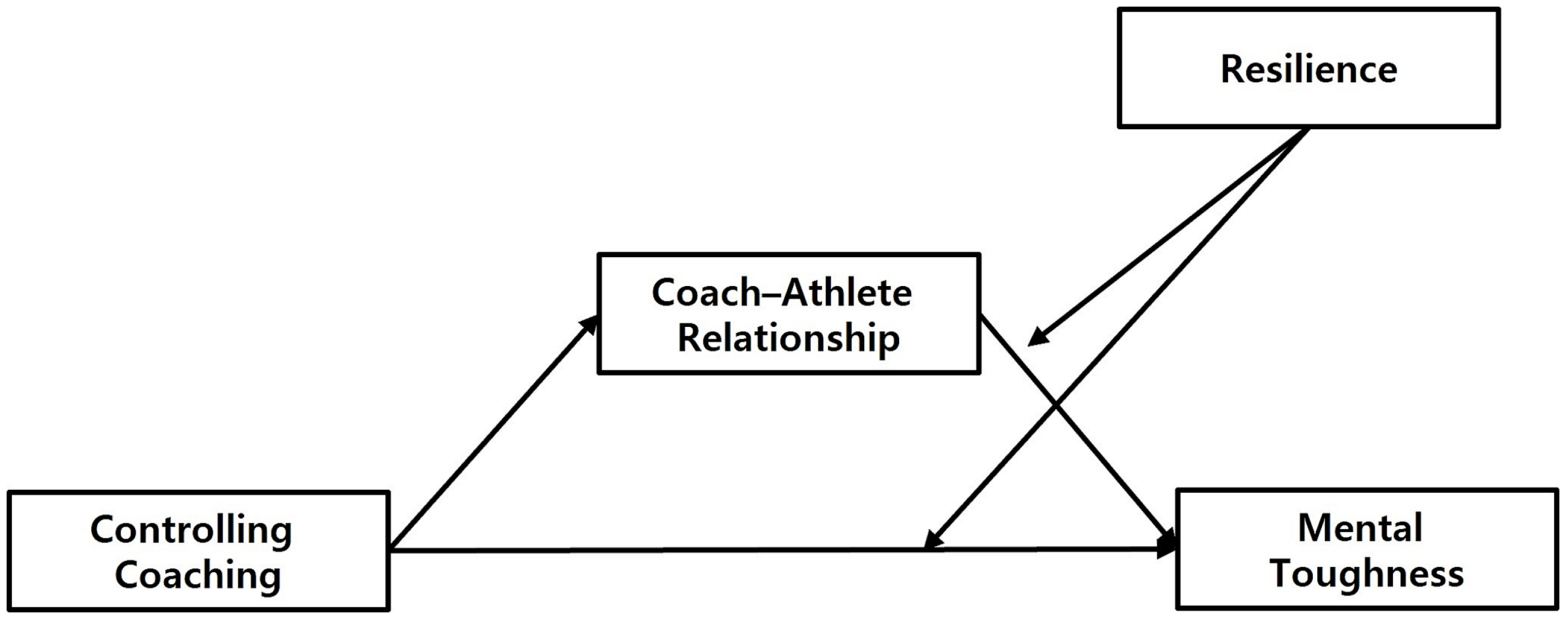
Moderated mediation model (aggregate level).

**Figure 2:**
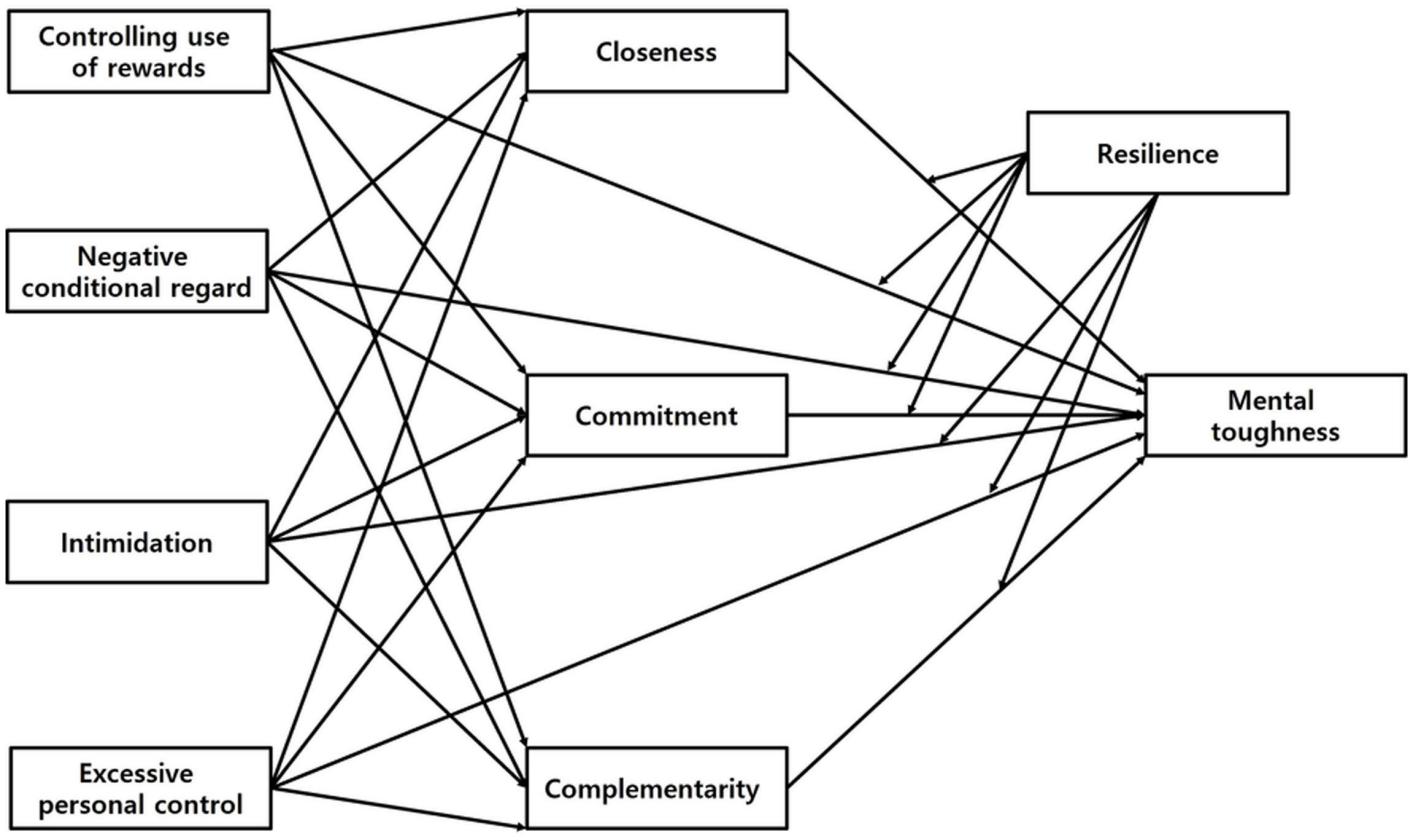
Moderated mediation model (sub-dimensional level).

Hypothesis 5: resilience will moderate the indirect effect of perceived controlling coaching behaviors on mental toughness through the CAR, such that the negative indirect effect will be significantly attenuated for athletes with higher levels of resilience (Figures 1, 2).

Hypothesis 5-1: Resilience will moderate the indirect effect of the specific facets of controlling coaching on mental toughness through the CAR sub-dimensions.

## 2 Methods

### 2.1 Research design and variable selection

To ensure methodological clarity and transparency, this study was grounded in a systematic literature review process conducted to refine our research framework. We adopted the PICO strategy (Santos et al., 2007) a conventional framework for structuring research questions and evidence searches to systematically organize the scope of our investigation based on peer-reviewed articles published between 2010 and 2026. Through databases such as Google Scholar and PsycINFO, we utilized keywords including “controlling coaching,” “coach-athlete relationship,” “resilience,” and “mental toughness” to identify consistent theoretical patterns. The resulting PICO components were defined as follows: the population (P) comprised elite collegiate athletes; the exposure (I) represented perceived controlling coaching behaviors; the comparison (C) focused on the differential effects across varying levels of resilience; and the outcome (O) addressed the maintenance of mental toughness. While this PICO-based systematic review structured our research inquiry, the conceptual model was further anchored in Self-Determination Theory (SDT) and the Coach-Athlete Relationship (CAR) model to provide a robust theoretical foundation for our hypotheses.

### 2.2 Participants and data collection

The participants of this study consisted of 206 active collegiate soccer players officially registered with the Korea Football Association (KFA). Registration with the KFA signifies that these athletes are elite competitors who undergo rigorous, professional-level training with the primary goal of transitioning into professional leagues. Thus, they represent a highly committed population facing significant performance-related and career-defining pressures.

Initially, 260 student-athletes were recruited through purposive and convenience sampling to ensure a representative sample of these elite competitors. Following the power analysis guidelines for regression-based models (Cohen, 1992; Green, 1991), a target of over 200 participants was established to secure sufficient statistical power for testing complex associations, including moderated mediation effects.

Data collection was conducted exclusively via an online survey platform. Participants were provided with a comprehensive briefing on the study’s objectives, the voluntary nature of their involvement, and the assurance of absolute anonymity. Informed consent was obtained electronically from all respondents prior to data entry. To ensure the integrity of the findings, a rigorous data screening process was applied; of the 260 responses collected, 55 were excluded due to incomplete data or evidence of insincere answering patterns (e.g., long-string responses). This resulted in a final sample of 205 valid responses for the main analysis. This study was conducted in accordance with ethical standards and received formal approval from the Institutional Review Board (IRB) of Dongguk University (Approval No. DUIRB-2026-04-21).

### 2.3 Measurements

#### 2.3.1 Controlling coaching behavior

To assess athletes’ perceptions of controlling coaching, the scale developed by Song and Cheon (2012) was utilized, which is grounded in the conceptual framework of Bartholomew et al. (2010, 2011). This instrument consists of 15 items evaluating four distinct sub-dimensions: controlling use of rewards, negative conditional regard, intimidation, and excessive personal control. While these dimensions represent specific facets of a controlling interpersonal style, we utilized a composite score for the primary analysis. This approach aligns with the empirical precedents established by the original scale developers (e.g., Bartholomew et al., 2011, 2018), who have employed a unidimensional composite score to represent the overall intensity of the controlling motivational climate. In the present study, the internal consistency (Cronbach’s *α*) for the total scale was 0.904. Furthermore, to support the subsequent sub-dimension level analyses, the reliability coefficients for each discrete facet were also evaluated and demonstrated robust internal consistency, with Cronbach’s α values of 0.866 for controlling use of rewards, 0.908 for negative conditional regard, 0.901 for intimidation, and 0.856 for excessive personal control.

#### 2.3.2 Coach–athlete relationship (CAR)

The quality of the CAR was measured using the 11-item Coach–Athlete Relationship Questionnaire (CART-Q), originally developed by Jowett (2007). This scale assesses the dyadic bond through three dimensions: closeness, commitment, and complementarity. Reflecting the methodological standards of the field, we calculated a composite score for the overarching moderated mediation model. This follows the procedures of Jowett and colleagues (e.g., Jowett, 2008, 2009; Jowett et al., 2023), who often aggregate these dimensions into a single relational quality index to facilitate a holistic assessment of the interpersonal dyad while maintaining theoretical parsimony. The Cronbach’s *α* for the total scale was 0.929. Consistent with the sub-dimension level analyses conducted in this study, the internal consistency for each latent construct was also examined, yielding Cronbach’s α values of 0.659 for commitment, 0.917 for closeness, and 0.894 for complementarity, all of which demonstrated satisfactory reliability for subsequent analyses.

#### 2.3.3 Resilience

Athletes’ psychological resilience was assessed using the 10-item Connor-Davidson Resilience Scale (CD-RISC-10), originally refined by Campbell-Sills and Stein (2007) from the original version by Connor and Davidson (2003), and validated in Korea by Cho (2014). This unidimensional scale measures the ability to adapt positively to stress and adversity (e.g., “I can deal with whatever comes my way”). Responses were recorded on a 5-point Likert scale (1 = Not at all to 5 = True nearly all the time). The internal consistency coefficient for resilience in this study was Cronbach’s alpha = 0.926.

#### 2.3.4 Mental toughness (MT)

To measure mental toughness, the study employed the Mental Toughness Index (MTI) developed by Gucciardi et al. (2015), with the Korean version validated by Han (2024). The MTI consists of 8 items that evaluate an athlete’s capacity to maintain purposeful and efficient performance under pressure (e.g., “I believe in my ability to achieve my goals”). Each item was rated on a 7-point Likert scale, ranging from 1 (Strongly Disagree) to 7 (Strongly Agree). The internal consistency of the MTI in the current sample was Cronbach’s alpha = 0.949.

### 2.4 Statistical analysis

To empirically examine the proposed research model, data processing was executed using IBM SPSS Statistics (v26.0) and AMOS (v26.0), alongside the PROCESS macro (v4.2). The analytical progression was structured as follows:

First, descriptive statistics and Pearson’s product–moment correlation analysis were performed to summarize the general characteristics of the sample. To address the multidimensionality of the core constructs as recommended by previous literature, these analyses were conducted for both the composite scores and the individual sub-dimensions of controlling coaching and the coach–athlete relationship. To assess the multivariate normality of the data, we examined the skewness and kurtosis of all focal variables. Following the recommendations of Kline (2015), all skewness values were within the absolute value of 2, and kurtosis values were below the absolute value of 7, indicating that the data did not significantly violate the assumption of a normal distribution.

Second, a Confirmatory Factor Analysis (CFA) was conducted using AMOS to evaluate the structural integrity of the measurement model. Reflecting the multidimensional structure of the scales, the CFA tested the fit of the hypothesized multi-factor structures (i.e., four factors for controlling coaching and three factors for the coach–athlete relationship) to ensure that each sub-dimension was psychometrically distinct. To verify the internal consistency and validity of the constructs, we calculated Composite Reliability (CR) and Average Variance Extracted (AVE). The model fit was assessed using multiple indices, including *χ*^2^/df, CFI, TLI, and RMSEA. Furthermore, to address potential Common Method Bias (CMB), Harman’s single-factor test was performed to ensure that a single factor did not account for the majority of the variance. To ensure the absence of multicollinearity before testing the structural model, we checked the Variance Inflation Factor (VIF) through multiple regression analysis, confirming that all values remained within acceptable thresholds (Figure 1).

Third, to examine the indirect effects of controlling coaching on mental toughness through the coach–athlete relationship (CAR), we first employed Hayes (2022) PROCESS macro Model 4. This baseline mediation analysis allowed us to verify the direct and indirect pathways without the influence of the moderator, providing a foundational understanding of the mediation mechanism (Figure 2).

Fourth, to test the sophisticated interplay of variables including the moderation effect of resilience, we utilized PROCESS macro Model 15. We initially analyzed composite scores for baseline patterns and subsequently performed primary analyses at the sub-dimension level to identify specific psychological mechanisms (Bartholomew et al., 2018; Jowett et al., 2023). Simple slope analysis was conducted to visualize the conditional impacts and indirect effects at distinct levels (±1 SD) of resilience. The statistical significance of the indirect effects and the Index of Moderated Mediation was determined using a bias-corrected bootstrapping procedure with 10,000 iterations.

## 3 Results

### 3.1 Demographic characteristics and preliminary analysis

The demographic profile of the participants consisted of 205 elite collegiate soccer players. The distribution of academic years was as follows: freshmen (*n* = 93, 45.4%), sophomores (*n* = 62, 30.2%), juniors (*n* = 37, 18.0%), and seniors (*n* = 13, 6.3%). To address the multidimensionality of the core constructs, descriptive statistics were calculated at the sub-dimension level. The mean scores for the four facets of controlling coaching were: 3.66 (SD = 1.52) for controlling use of rewards, 3.19 (SD = 1.48) for negative conditional regard, 2.52 (SD = 1.36) for intimidation, and 2.10 (SD = 1.24) for excessive personal control. In line with previous research (e.g., Bartholomew et al., 2018), a composite mean score for controlling coaching was also computed (*M* = 2.92, SD = 1.06). Regarding the coach–athlete relationship, the mean scores for the sub-dimensions of closeness, commitment, and complementarity were 4.10 (SD = 0.80), 3.59 (SD = 0.77), and 4.15 (SD = 0.73), respectively, with a composite mean score of 3.98 (SD = 0.69). The mean scores for resilience and mental toughness were 3.98 (SD = 0.71) and 5.85 (SD = 1.00), respectively. To evaluate the assumption of multivariate normality, the skewness and kurtosis of all focal variables and their respective sub-dimensions were examined. Following the criteria proposed by Kline (2015), the skewness values for all variables ranged from −0.769 to 1.004, and the kurtosis values ranged from −0.887 to 0.703. These results confirm that the data do not significantly deviate from a normal distribution, as all values remained well within the absolute thresholds of 2.0 for skewness and 7.0 for kurtosis. The comprehensive descriptive statistics and normality indicators are presented in Table 1.

**Table 1:** Descriptive statistics.

Variables	Mean	SD	Skewness	Kurtosis
Controlling coaching	2.92	1.06	0.053	−0.606
- Controlling use of rewards	3.67	1.53	0.048	−0.391
- Negative conditional regard	3.19	1.49	0.073	−0.887
- Intimidation	2.53	1.37	0.657	−0.496
- Excessive personal control	2.11	1.25	1.004	0.024
Coach–athlete relationship	3.98	0.69	−0.578	0.371
- Commitment	3.59	0.77	−0.291	0.203
- Closeness	4.11	0.80	−0.748	0.314
- Complementarity	4.15	0.73	−0.769	0.703
Resilience	3.98	0.71	−0.397	−0.297
Mental toughness	5.85	1.01	−0.73	−0.224

Pearson’s correlation analysis revealed statistically significant associations among all focal variables in the hypothesized directions (*p* < 0.01). As presented in Table 2, the sub-dimensional level, all four facets of controlling coaching (i.e., controlling use of rewards, negative conditional regard, intimidation, and excessive personal control) exhibited significant negative correlations with the three dimensions of the coach–athlete relationship (i.e., commitment, closeness, and complementarity) and mental toughness. Notably, excessive personal control (*r* = −0.364) and intimidation (*r* = −0.354) showed the most pronounced negative associations with mental toughness. Regarding the relational aspects, commitment (*r* = 0.473), closeness (*r* = 0.469), and complementarity (*r* = 0.576) were significantly and positively linked to mental toughness. To maintain theoretical alignment with prior research (Bartholomew et al., 2018; Jowett et al., 2023), composite scores were also computed; the overall controlling coaching score was negatively correlated with the overall coach–athlete relationship (*r* = −0.532), resilience (*r* = −0.296), and mental toughness (*r* = −0.371). Correlations among sub-dimensions within each construct (ranging from 0.502 to 0.865 for controlling coaching and 0.662 to 0.820 for the coach–athlete relationship) suggest that while these facets are theoretically related, they maintain sufficient statistical distinctiveness. Finally, multicollinearity was assessed using Variance Inflation Factor (VIF) at the sub-dimension level; VIF values ranged from 1.095 to 3.490, well below the acceptable threshold of 5.0, thereby supporting the validity of the subsequent moderated mediation analysis.

**Table 2:** Correlation matrix, reliability, and convergent validity.

Variables	1	2	3	4	5	6	7	8	9	10
1. CC	1									
2. CC1	−0.532^**^	1								
3. CC2	−0.296^**^	0.491^**^	1							
4. CC3	−0.371^**^	0.560^**^	0.778^**^	1						
5. CC4	0.600^**^	−0.167^*^	−0.068	−0.169^*^	1					
6. CAR	0.803^**^	−0.529^**^	−0.287^**^	−0.269^**^	0.201^**^	1				
7. CAR1	0.865^**^	−0.512^**^	−0.297^**^	−0.354^**^	0.255^**^	0.694^**^	1			
8. CAR2	0.757^**^	−0.414^**^	−0.264^**^	−0.364^**^	0.240^**^	0.502^**^	0.716^**^	1		
9. CAR3	−0.394^**^	0.838^**^	0.451^**^	0.473^**^	−0.066	−0.466^*^	−0.369^*^	−0.296^*^	1	
10. Resilience	−0.519^**^	0.933^**^	0.415^**^	0.469^**^	−0.177^*^	−0.496^*^	−0.508^*^	−0.397^*^	0.662^*^	1
11. MT	−0.512^**^	0.930^**^	0.472^**^	0.576^**^	−0.189^*^	−0.470^*^	−0.492^*^	−0.413^*^	0.674^*^	0.820^**^

**p < 0.01; CC, controlling coaching; CAR, coach–athlete relationship; MT, mental toughness. For controlling coaching, CC1–CC4 represent the sub-dimensions of controlling use of rewards, negative conditional regard, intimidation, and excessive personal control, respectively; CAR1–CAR3 denote closeness, commitment, and complementarity.

### 3.2 Measurement model analysis

To evaluate the structural integrity and factorial validity of the instruments, a Confirmatory Factor Analysis (CFA) was performed. The measurement model was specified as a multi-factor structure, where the sub-dimensions of controlling coaching (four factors) and the coach–athlete relationship (three factors) were treated as distinct first-order factors. This approach was adopted to respect the multidimensional nature of the constructs and to ensure that each facet was psychometrically distinguished. As summarized in Table 3, the hypothesized multi-factor model demonstrated an acceptable fit (χ^2^ = 2.075, CFI = 0.924, TLI = 0.915, RMSEA = 0.073, SRM*R* = 0.052). To address potential common method bias (CMB), we compared this hypothesized model with a single-factor model, in which all indicators were constrained to load onto a single latent factor (Podsakoff et al., 2003). The single-factor model exhibited a very poor fit (χ^2^ = 4.766, CFI = 0.729, TLI = 0.704, RMSEA = 0.136, SRMR = 0.098), suggesting that CMB was not a pervasive concern in the current study and further confirming the discriminant validity of the multi-dimensional structure (Table 4).

**Table 3:** Results of Confirmatory Factor Analysis for Model Comparison.

Model	χ^2^/df	CFI	TLI	RMSEA	SRMR
multi-factor model	2.075	0.924	0.915	0.073	0.052
single-factor model	4.766	0.729	0.704	0.136	0.098

**Table 4:** Psychometric properties of the measurement model.

Variables	Items/factors	Loading range	CR	AVE	√AVE
Controlling coaching	4	0.280–0.940	0.769	0.506	0.711
(1) Controlling use of rewards		0.280			
(2) Negative conditional regard		0.736			
(3) Intimidation		0.940			
(4) Excessive personal control		0.752			
Coach–athlete relationship	3	0.738–0.926	0.886	0.724	0.851
(1) Closeness		0.884			
(2) Commitment		0.738			
(3) Complementarity		0.926			
Mental Toughness	8	0.776–0.872	0.951	0.710	0.843
Resilience	10	0.658–0.822	0.926	0.558	0.747

CR, composite reliability; AVE, average variance extracted.

The standardized factor loadings for the parcels ranged from 0.280 to 0.940. Specifically, for controlling coaching, the loading for ‘controlling use of rewards’ was relatively low (0.280); however, the overall construct reliability (CR = 0.769) and AVE (0.506) remained above the recommended thresholds of 0.70 and 0.50, respectively (Hair et al., 2010), justifying its retention as a theoretically significant facet.

Furthermore, the AVE values for coach–athlete relationship (0.724), mental toughness (0.710), and resilience (0.558) were well above the suggested threshold of 0.50, establishing robust convergent validity. For the controlling coaching construct, the AVE was 0.506, which also met the minimum requirement. To establish discriminant validity, we compared the square root of the AVE for each construct with the correlation coefficients between that construct and all others (Fornell and Larcker, 1981). The results indicated that the square root of the AVE for each latent variable (ranging from 0.711 to 0.851) was consistently greater than its correlations with any other variables in the model. This confirms that each construct captures unique variance and is empirically distinct from the other constructs. Collectively, these results demonstrate that the measurement model possesses adequate reliability, convergent validity, and discriminant validity, providing a solid foundation for testing the structural relationships among the variables.

### 3.3 Hypothesis testing

#### 3.3.1 Direct and mediating effects

To verify the proposed hypotheses, we conducted an integrated analysis using Hayes (2022) PROCESS macro (Model 4). The analysis proceeded in two stages: an aggregate model using composite scores to establish baseline psychological mechanisms, and a sub-dimensional model to dissect the specific behavioral facets driving these dynamics (see Table 5).

**Table 5:** Direct and indirect effects of controlling coaching on mental toughness (model 4).

Predictor	Direct effect	Indirect effect			
		Total	Commitment	Closeness	Complementarity
Controlling coaching	−0.096	−0.255*	-	-	-
Controlling use of rewards	−0.038	−0.009	0.006	0.002	−0.017
Negative conditional regard	0.092	−0.119*	−0.057*	0.015	−0.077*
Intimidation	−0.044	−0.068	−0.008	0.016	−0.077
Excessive personal control	−0.126	−0.05	−0.011	0.004	−0.043

*p < 0.05.

Regarding Hypothesis 1 and 1–1, we examined the direct association between controlling coaching and mental toughness. In the aggregate model, controlling coaching exhibited a marginal direct negative association with mental toughness (*β* = −0.096, *p* = 0.141). At the sub-dimensional level, although no individual facet reached statistical significance, ‘excessive personal control’ (*β* = −0.126) and ‘intimidation’ (*β* = −0.044) emerged as the facets exerting the most pronounced negative pressure, suggesting that the direct inhibitory effect of controlling behaviors is subtle and distributed across various behavioral dimensions.

Regarding Hypothesis 2 and 2–1, we examined the mediating role of the coach–athlete relationship (CAR). The aggregate model confirmed a significant indirect effect [*β* = −0.255, 95% CI (−0.356, −0.166)], where controlling coaching was negatively associated with the CAR, which in turn served as a critical psychological resource for mental toughness. At the sub-dimensional level, the analysis delineated that ‘negative conditional regard’ was the primary driver of this mediation effect [*β* = −0.119, 95% CI (−0.196, −0.052)]. Specifically, this facet undermined athletes’ commitment (*β* = −0.057) and complementarity with the coach (*β* = −0.077), thereby indirectly attenuating their mental toughness. While other facets (e.g., ‘intimidation’ and ‘excessive personal control’) showed directional trends in undermining relational quality, ‘negative conditional regard’ emerged as the most potent behavioral facet that compromises the relational foundation of an athlete’s mental fortitude.

#### 3.3.2 Testing for moderated mediation

To test the proposed moderation and moderated mediation hypotheses (H3–H5), we examined the conditional effects within the Model 15 framework. Regarding Hypothesis 3 and 3–1, resilience did not moderate the association between controlling coaching and the CAR (*β* = 0.026, *p* = 0.676). This null finding was consistent across both the aggregate and sub-dimensional models, suggesting that the deleterious impact of controlling coaching on the coach–athlete relationship is pervasive regardless of an athlete’s resilience.

Regarding Hypotheses 4 and 4–1, we found that resilience significantly moderated the second-stage path (CAR → mental toughness; *β* = −0.271, *p* < 0.01). The addition of this interaction term accounted for significant incremental variance (Δ*R*^2^ = 0.0122, *p* < 0.01). The Johnson-Neyman technique revealed that the positive link between CAR and mental toughness was significant only for athletes with lower-to-average resilience (below +0.35 SD). For highly resilient athletes, this link was non-significant, suggesting that resilience functions as a psychological buffer that sustains mental fortitude even when the coach–athlete relationship is suboptimal.

Finally, regarding Hypotheses 5 and 5–1, the moderated mediation analysis confirmed a significant second-stage conditional indirect effect [Index = 0.0943, 95% Boot CI (0.0249, 0.1691)]. As presented in Table 6, the negative indirect effect of controlling coaching on mental toughness was significant at low [Effect = −0.166, 95% CI (−0.255, −0.076)] and average levels of resilience [Effect = −0.091, 95% CI (−0.158, −0.026)], whereas this negative impact was effectively neutralized at high levels of resilience [Effect = −0.006, 95% CI (−0.096, 0.080)]. Regarding the sub-dimensional analysis, the index of moderated mediation was not statistically significant for all facets. However, conditional effect analysis showed the following: ‘negative conditional regard’ had a direct negative impact on mental toughness for athletes with lower levels of resilience (*β* = 0.1192, *p* = 0.0378). Additionally, the indirect path from ‘negative conditional regard’ through ‘complementarity’ to mental toughness was statistically significant for athletes with low to average resilience [*β* = −0.052, 95% CI (−0.126, −0.006)].

**Table 6:** Conditional indirect effects at levels of resilience.

Resilience level	Effect	SE	Boot LLCI	Boot ULCI
Low (−1 SD)	−0.166	0.046	−0.255	−0.076
Average (Mean)	−0.091	0.034	−0.158	−0.026
High (+1 SD)	−0.006	0.045	−0.096	0.08
Index of Moderated Mediation	0.094	0.036	0.025	0.169

Bootstrapping iterations = 5,000; LLCI, lower limit confidence interval; ULCI, upper limit confidence interval. Indirect effect is significant when the 95% Boot CI does not include zero.

## 4 Discussion

The primary objective of this study was to examine an integrated moderated mediation model to delineate how perceived controlling coaching, the coach–athlete relationship, and individual resilience collectively relate to the mental toughness of elite collegiate soccer players. The results provide nuanced evidence that while environmental stressors associated with the “dark side” of coaching are linked to diminished psychological fortitude, individual adaptive resources serve as a critical boundary condition that can neutralize these negative associations.

### 4.1 The direct and relational fallout of controlling coaching

The negative association between perceived controlling coaching and CAR quality supports Hypothesis 2 and aligns with Self-Determination Theory (SDT). SDT posits that optimal psychological growth depends on satisfying the innate needs for autonomy, competence, and relatedness (Deci and Ryan, 2000; Ryan and Deci, 2017). Coercive interpersonal styles act as significant need-thwarting catalysts (Bartholomew et al., 2011). When athletes perceive their environment as controlling, their sense of autonomy is violated, leading to a deterioration of the relational bond with the coach. Extending this to Hypothesis 2–1, sub-dimensional analysis reveals that negative conditional regard is the primary driver of relational decay, specifically undermining commitment and complementarity. This indicates that behaviors involving the conditional withdrawal of support are particularly toxic to the affective and cognitive bonds of the coach-athlete dyad.

Regarding the direct association between controlling coaching and mental toughness (Hypothesis 1), while significant in preliminary correlations, its predictive power attenuated in the multivariate model. In line with Hypothesis 1–1, sub-dimensional results showed that excessive personal control and intimidation exerted the most pronounced negative pressure; however, the lack of statistical significance across these facets suggests that coaching’s dark side (Mageau and Vallerand, 2003) primarily impacts the athlete’s psyche through complex relational channels rather than direct pathways. Consistent with Jowett’s (2007) 3 + 1Cs framework, these behaviors erode closeness, commitment, and complementarity. In the high-stakes environment of collegiate soccer, where the coach-athlete dyad represents the primary social context (Bull et al., 2005), coercive power destabilizes this relational foundation (Jowett and Poczwardowski, 2007). Consequently, the athlete’s capacity for challenge appraisal is impeded, eroding the core components of mental toughness (Jones et al., 2007; Rodahl et al., 2015).

### 4.2 Limits of individual buffering on relational stress

Contrary to Hypothesis 3 and 3–1, resilience did not significantly moderate the association between perceived controlling coaching and the CAR. This finding suggests that within the rigid hierarchical and result-oriented culture of elite collegiate athletics, a coach’s controlling behavior represents an objective environmental stressor of such magnitude that individual psychological traits cannot easily mitigate its impact at the relational stage. As established in the introduction, the coach-athlete dyad in the collegiate setting often functions as the most influential hierarchical relationship, where the coach serves as the primary architect of the athlete’s psychological environment (Bull et al., 2005; Gucciardi et al., 2015). Given the dual-career framework characterized by professional scrutiny and vocational instability (Stenling and Tafvelin, 2014; Gustafsson et al., 2017), the use of coercive power is likely perceived not as a subjective challenge to be managed, but as a definitive threat to the athlete’s professional viability.

Furthermore, while resilience is conceptualized as a dynamic process of positive adaptation (Luthar et al., 2000; Masten, 2011), the present results indicate that its protective capacity has limits when confronted with the dark side of coaching. Specifically, sub-dimensional testing confirmed that even highly resilient athletes could not prevent the erosion of the coach-athlete relationship when faced with systemic controlling behaviors. This consistency across all facets, from controlling use of rewards to privacy interference, underscores the dominance of the coach’s authority in elite sectors. Regardless of an athlete’s resilience, restrictive interpersonal styles appear to invariably diminish the perceived quality of the dyadic bond, rendering even the most resilient athletes vulnerable to need-thwarting behaviors (Deci and Ryan, 2000).

The lack of moderation at this stage supports the perspective that certain environmental risk factors in elite sports are so systemic and intrusive that they may temporarily overwhelm individual protective factors (Amorose and Anderson-Butcher, 2007; Stebbings et al., 2012). It implies that resilience, rather than acting as a preventative shield that stops the relationship from deteriorating, may only begin to function effectively once the relational damage has occurred. This distinction is crucial, as it suggests that while internal assets are vital for self-regulation, they cannot serve as a substitute for a supportive social context; the structural fallout of controlling coaching remains a potent interpersonal reality that resilience alone cannot neutralize before it erodes the coach-athlete bond (Jowett, 2017).

### 4.3 Resilience as a psychological firewall

A pivotal discovery of this research is the significant moderating role of resilience in the association between the CAR and mental toughness, supporting Hypothesis 4 and 4–1. The Johnson-Neyman (J-N) analysis yielded a sophisticated boundary condition: the CAR remained a significant predictor of MT only for athletes whose resilience scores were below the threshold of 0.351 (mean-centered), representing approximately 66.34% of the participants. For the upper 33.66% of highly resilient athletes, the link was non-significant.

This finding identifies resilience as a psychological firewall that facilitates a state of relational independence. The granular examination of this moderation reveals that resilience is particularly effective in anchoring mental toughness when the complementarity dimension of the relationship is compromised. This suggests that resilient athletes maintain their professional and mental fortitude through internal self-regulatory processes, even when the functional coordination with their coach is suboptimal. While the introduction established that the CAR typically functions as a primary relational conduit for an athlete’s developmental experiences (Jowett and Poczwardowski, 2007), these results suggest that such reliance is not universal. For athletes with lower resilience, the quality of the bond with the coach remains a non-negotiable prerequisite for mental fortitude; for them, a weakened relationship likely triggers a “threat appraisal,” which erodes the internal resources required to persist through adversity (Mahoney et al., 2014; Jones et al., 2007).

In contrast, highly resilient athletes possess superior cognitive flexibility, allowing them to engage in positive reappraisal even in suboptimal interpersonal environments (Sarkar and Fletcher, 2014). In the collegiate setting—a decisive phase where vocational viability is scrutinized (Stenling and Tafvelin, 2014)—these athletes decouple their internal fortitude from suboptimal contexts. Rather than viewing relational friction as an insurmountable threat to their identity or vocational future, they interpret it as a manageable external hurdle. This decoupling mechanism allows highly resilient athletes to neutralize the systemic fallout of a compromised CAR, ensuring that their mental toughness remains an anchored asset.

### 4.4 The neutralizing mechanism: moderated mediation

The statistical significance of the index of moderated mediation serves as the empirical culmination of this study, confirming Hypothesis 5 and 5–1. These results demonstrate that the negative indirect association of controlling coaching on mental toughness, as mediated by the coach–athlete relationship, is fundamentally contingent upon the athlete’s internal adaptive profile. For the majority of athletes specifically those exhibiting low to average resilience the pathway of psychological erosion remains highly permeable. In these instances, the relational damage inflicted by a controlling coach directly translates into a depletion of the athlete’s mental fortitude, confirming that without sufficient internal buffers, the systemic fallout of a coercive environment is near-inevitable (Bartholomew et al., 2011; Occhino et al., 2014).

The most theoretically salient finding, however, is the neutralization of this indirect link at high levels of resilience (+1 SD). Our findings indicate that “negative conditional regard” and “intimidation” consistently drive this pathway of psychological erosion, yet high resilience effectively acts as a “circuit breaker” in the second stage of the process. These results advance the social-ecological perspective of mental toughness (Connaughton et al., 2008) by illustrating that resilience functions not merely as a trait, but as a dynamic “circuit breaker” within dysfunctional sporting environments. While the rejection of Hypothesis 3 indicated that resilience cannot necessarily prevent the coach from adopting controlling behaviors or the relationship from suffering initial decay, the moderated mediation results prove that resilience effectively inhibits this relational decay from “bleeding” into the athlete’s ultimate psychological edge (Anyan and Hjemdal, 2016; Wagstaff et al., 2017).

This boundary effect suggests a sophisticated mechanism of psychological risk management (Stebbings et al., 2012). For highly resilient athletes, the negative trajectory from the dark side of coaching (Mageau and Vallerand, 2003) to compromised performance mindsets is intercepted at the second stage of the mediation process. By mobilization of superior self-regulatory capacities and the effective use of psychological resources (Fletcher and Sarkar, 2013), these athletes maintain their purposeful and efficient functioning despite the presence of interpersonal adversity. Consequently, high resilience serves as a critical decisive threshold where controlling coaching despite its pervasive presence in elite sectors loses its empirical capacity to compromise the core psychological determinants of objective performance outcomes. This finding provides a comprehensive map of how mental toughness is sustained, demonstrating that the interplay between external environmental architects and internal adaptive assets defines the ultimate differentiation in an athlete’s vocational viability.

### 4.5 Practical implications and limitations

While the present findings provide critical insights into the protective mechanisms of resilience, several methodological constraints must be acknowledged. First, the cross-sectional design of this study precludes the establishment of strict causal inferences. Although the moderated mediation model was grounded in robust theoretical frameworks such as SDT and Interdependence Theory, the observed associations represent a single temporal snapshot. Future research should utilize longitudinal or diary-study designs to capture the dynamic fluctuations in coaching behaviors and athlete responses over a competitive season, thereby elucidating the long-term cumulative impact of controlling environments on psychological growth. Furthermore, the reliance on self-report measures introduces the possibility of common-method bias; integrating multi-source data, such as coach-rated performance or objective physiological markers of stress, would enhance the empirical rigor of subsequent investigations.

Despite these limitations, the practical utility of this study for elite collegiate sports is substantial. From a coaching education perspective, the relational dependence observed in 66% of the sample suggests that the widespread use of controlling strategies as a means of ensuring compliance is a high-risk gamble. In the critical developmental juncture of collegiate athletics, such styles do not merely diminish immediate well-being but potentially compromise the very vocational viability (Stenling and Tafvelin, 2014) of the majority of athletes who lack the internal “firewall” to withstand interpersonal friction. Our sub-dimensional analysis specifically highlights that negative conditional regard is the primary relational toxin; thus, coaching education must explicitly emphasize the detrimental impact of conditional support on relational bonds. Therefore, sports organizations should prioritize training programs that facilitate autonomy-supportive coaching, emphasizing that the coach-athlete relationship is the primary infrastructure for mental fortitude.

From a psychological risk-management standpoint, these results advocate for a dual-track intervention strategy. While environmental reform is paramount, the structural embeddedness of coaching styles in elite soccer often makes immediate systemic change difficult. Consequently, sport psychology practitioners should implement Resilience Training Programs (RTPs) focused on cognitive restructuring and positive reappraisal (Sarkar and Fletcher, 2014). By equipping athletes with the capacity to decouple their self-evaluation from the coach’s interpersonal fluctuations, practitioners can provide a secondary layer of protection—a psychological insurance policy—that ensures mental toughness remains anchored even when the social context is suboptimal. Future research should specifically investigate the efficacy of such interventions in “neutralizing” the longitudinal fallout of controlling coaching, potentially exploring other moderators such as trait mindfulness or perceived organizational support as additional layers of the psychological firewall.

## 5 Conclusion

In conclusion, this study demonstrates that while controlling coaching behaviors are negatively associated with mental toughness through the erosion of the coach–athlete relationship, this destructive pathway is not universal. The findings highlight resilience as a critical decisive threshold; it functions as a psychological firewall that decouples an athlete’s internal fortitude from interpersonal stressors. Specifically, the sub-dimensional analysis revealed that behaviors such as negative conditional regard serve as primary drivers of relational decay, yet high resilience effectively neutralizes their impact. For the most resilient athletes, the negative impact of a compromised relational bond is effectively neutralized, allowing for the maintenance of mental toughness even in suboptimal social contexts. Ultimately, these results underscore that protecting an athlete’s vocational viability in elite sports requires a dual focus on fostering autonomy-supportive environments and proactively developing individual adaptive resources.

## Data Availability

The raw data supporting the conclusions of this article will be made available by the authors, without undue reservation.
